# RBE-Guided Treatment Planning, LET Optimization, and Implications of Proton Arc Therapy for the Sparing of Nervous Tissue in Head and Neck Proton Therapy

**DOI:** 10.3390/cancers17233724

**Published:** 2025-11-21

**Authors:** Keaton Reiners, Mark Artz, Curtis M. Bryant, Roi Dagan, Hardev S. Grewal, Perry B. Johnson, Zuofeng Li, Jiyeon Park, Michael Vieceli, Yawei Zhang

**Affiliations:** 1 UF Health Proton Therapy Institute, Jacksonville, FL 32206, USA; 2 Medical Physics Graduate Program, University of Florida College of Medicine, Gainesville, FL 32610, USA; 3 Southwest Florida Proton, Fort Meyers, FL 33967, USA; 4 Department of Radiation Oncology, University of Florida College of Medicine, Gainesville, FL 32610, USA

**Keywords:** PBS LET optimization, RBE model dose, proton dynamic arc, head and neck

## Abstract

**Background/Objectives**: Traditionally, proton therapy assumes a fixed relative biological effectiveness (RBE) value of 1.1, which can lead to inaccurate dose calculations in treatment planning. This study examines the effect of dynamic arc delivery and pencil beam scanning (PBS) linear energy transfer (LET) optimization on LET-dependent RBE dose escalation to nervous tissue structures in head and neck (H&N) cancer patients undergoing proton beam therapy, utilizing two RBE dose models. **Methods**: Fifteen head and neck cancer patients previously treated with PBS proton therapy at high risk of nervous tissue toxicity were retrospectively analyzed. Three plans were developed for each patient: PBS, dynamic arc, and PBS with LET optimization. RBE-weighted dose distributions were calculated and compared for all patient plans using a linear LET-weighted model and a tissue-specific α/β-dependent model. LET-dose (LET_d_) constraints were systematically tested to determine optimal values for plan quality and efficacy in altering LET spatial distribution in regions of concern. LET-dependent RBE enhancement was calculated for the three different planning methods. **Results**: Dynamic arc plans increased RBE enhancement when compared to PBS, while LET optimization for PBS consistently reduced RBE-enhanced dose to nervous tissue compared to non-optimized plans for both RBE models. Patient-specific variability in optimization benefit was observed, with the most significant improvements in cases with greater initial RBE enhancement. A maximum LET_d_ constraint of 2.5 µm/keV above 80% of the maximum structure dose threshold was found to balance plan quality and RBE mitigation. **Conclusions**: Dynamic arc delivery increased RBE enhancement relative to static PBS. LET optimization successfully modified LET_d_ spatial distributions to minimize RBE enhancement to nervous tissue structures when compared to non-LET optimized PBS and dynamic arc plans. Patient-specific risk stratification should be used when clinically deploying LET optimization.

## Simple Summary

The dose calculation within a treatment planning system for proton therapy has historically been performed with an assumed RBE of 1.1. This assumption does not account for heterogenous LET distributions and therefore the changes to dose distribution resulting from LET-dependent RBE dose. With LET optimization becoming commercially available within treatment planning systems, there is a demand for exploring the impacts of LET distributions, dosimetric consequences, and optimization methodology. This work explores the impact of LET on RBE dose, as well as the impact of beam delivery method–PBS and dynamic arc–on LET distributions to nervous tissue critical structures in head and neck cancer patients. Furthermore, the study provides clinical recommendations for the clinical deployment of LET optimization.

## 1. Introduction

Proton beam therapy (PBT) is increasingly being deployed in the treatment of head and neck cancers due to its ability to deliver high-dose radiation with superior spatial precision, greater dose conformality, and the potential to minimize radiation-induced toxicities to surrounding healthy tissues and critical structures compared to photon-based modalities [1,2,3,4,5]. This advantage primarily stems from the spread-out Bragg peak (SOBP) phenomenon, which allows protons to deposit most of their dose at a well-defined tissue depth by modulating the proton beam’s energy [6,7,8].

Despite the dosimetric benefits, a persistent challenge remains in accurately accounting for and quantifying the relative biological effectiveness (RBE) of protons. RBE, which reflects the enhanced biological effectiveness of protons compared to photons, varies along the proton path and is typically highest at the distal edge of the Bragg peak [9,10,11]. Historically, treatment planning systems (TPS) have assumed a constant RBE value of 1.1, a simplification that may underestimate the actual biological dose delivered to adjacent healthy tissue and critical structures, potentially leading to unanticipated toxicities and adverse effects [12,13,14,15,16,17].

This oversimplification overlooks the impact of linear energy transfer (LET). This physical parameter is defined as the energy deposited per unit length of tissue and strongly correlates with biological damage mechanisms, such as single-strand and double-strand DNA breakage [18,19,20]. LET increases toward the distal edge of the Bragg peak, mirroring the rise in RBE, and has therefore been proposed as a surrogate for biologically informed treatment planning [9,10]. Multiple studies have demonstrated a spatial relationship between regions of high LET and increased risk of radiation-induced toxicities, including necrosis, mucositis, brainstem injury, brachial plexopathy, and osteoradionecrosis [16,17,21,22]. These effects are especially relevant in head and neck cancers, where tumor volumes often lie close to critical structures/OARs such as the optic chiasm and brainstem. Given the difficulty of implementing variable RBE models clinically, LET-based optimization has emerged as a pragmatic alternative due to model dependency and a lack of consensus. LET can be accurately calculated through Monte Carlo simulations or analytical methods and incorporated into robust inverse planning frameworks [10,23,24,25].

Advanced strategies, such as LET-weighted dose optimization, biological surrogate dose, and LET painting, aim to manipulate the spatial distribution of LET to move high-LET areas away from OARs and into the tumor target [9,26,27,28]. Beyond software and computational-based improvement techniques to LET distribution, various beam delivery methods within intensity-modulated proton therapy (IMPT) are being explored. For instance, in pencil beam scanning (PBS) proton therapy, LET distributions can be modulated by adjusting beam angles, spot sizes, and energy layer spacing. This enables a degree of biological control that is not available in passive scattering approaches. PBS-based LET optimization has been shown to reduce LET hotspots within sensitive structures while preserving tumor coverage [9,27,29]. These strategies are supported by studies that show potential clinical benefits of LET-guided planning have that link adverse effects to elevated LET distributions, as well as dosimetric investigations showing that high LET can occur in low physical dose regions, especially near the edges of treatment fields, leading to underestimation of biological risk and making RBE 1.1 dose-only review of plans potentially misleading [20,30,31,32]. Beyond PBS, a newer, emerging beam delivery technique within PBT/IMPT is proton arc therapy (PAT), a dynamic modality that involves continuous gantry rotation, further complicating the landscape. While this modality offers potential improvements in dose conformality, the literature exploring its effects on LET and corresponding RBE alterations is currently sparse [33,34,35]. Early planning studies suggest that PAT may increase low-dose volume and LET heterogeneity, potentially exacerbating the biological dose to nearby OARs [35]. While dosimetric benefits have been demonstrated in complex anatomical regions, the LET implications of PAT remain understudied. The dynamic nature of arc delivery introduces greater overlap of distal ends of proton paths, which may concentrate high LET in unintended regions. This highlights an urgent need to investigate how LET behaves under arc-based delivery.

This study aims to address several key gaps in the current literature by pursuing three primary objectives:

• Analyze the effect of LET on RBE for head and neck cancer patients, focusing on LET-dependent RBE dose to nervous tissue.
• Explore the implications and impact of proton dynamic arc therapy on spatial LET distributions compared to conventional PBS.
• Establish clinically feasible recommendations for the use of LET optimization in head and neck proton therapy by finding optimal LET optimization parameters to improve treatment safety and efficacy.

By addressing these aims, we seek to establish further and highlight how LET-guided planning may serve as a critical bridge that links the physical precision of with the biological uncertainties of tissue response in PBT.

## 2. Materials and Methods

### 2.1. Patient Selection

Fifteen patients with head and neck cancer were retrospectively enrolled in this study under a protocol (UFPTI2210-HNX08) approved by the institutional review board at the University of Florida. All patients were previously treated on a Proteus^®^ONE PBS gantry (Ion Beam Applications SA, Louvain-la-Neuve, Belgium) between January 2020 and January 2022. Patients were selected based on one primary criterion: if the patient had a maximum physical dose to the brainstem, optic chiasm, optic nerves, or brachial plexus of 50 Gy or more. At this physical dose, patients are at risk of exceeding the maximum dose allowed to these structures, as determined by an LET-based RBE model dose calculation. This maximum dose was set at 60 Gy, based on dose restraints suggested in the QUANTEC reports [36]. Most patients who met this criterion had target volumes near the nasopharyngeal region, except those with high brachial plexus doses, as these patients had disease that extended lower down the neck.

### 2.2. Treatment Plan Design & Prescription

**Figure 1:**
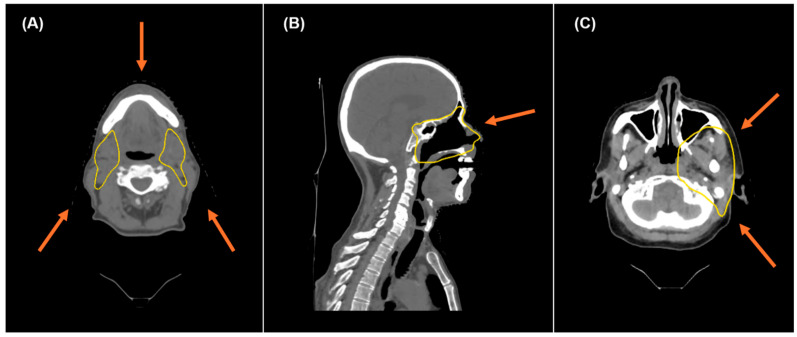
(**A**) provides an axial view of the common beam arrangement used for bilateral disease, while (**B**) provides a sagittal view of the anterior–posterior beam angle. (**C**) demonstrates the typical beam arrangement used for unilateral disease. The standard-risk CTV is shown with a yellow contour for the bilateral and unilateral cases.

While head and neck target sites vary widely, treatment planning was kept as consistent as possible, adhering to our institution’s planning standards. The beam geometry and contours were not modified from the initial plan used for the patient’s treatment. The most common beam arrangement for bilateral disease was a 3-beam arrangement, shown in Figure 1A, with one anterior–posterior beam and two posterior oblique beams, or one posterior–anterior beam with two anterior oblique beams. For unilateral disease, the most common beam arrangement was a 2-beam approach, shown in Figure 1C, with an anterior oblique and posterior oblique beam.

**Figure 2:**
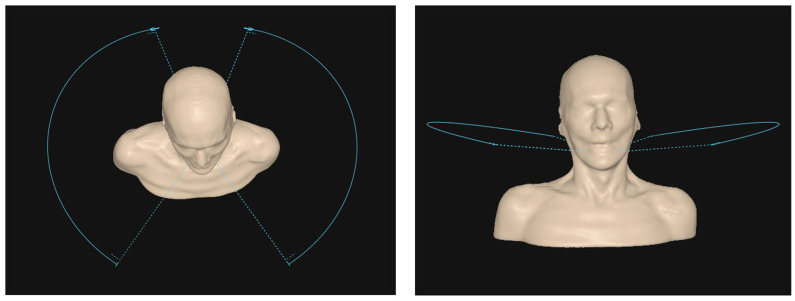
The left image illustrates the common arc beam arrangement for bilateral disease, while the right image demonstrates the additional 10° couch kick used for both arc and static beams to avoid treating through the shoulders when the disease extends lower.

For proton arc plans, a typical arrangement was two 130° arcs, from 30 to 160°, as shown in Figure 2, with a 180° couch kick, resulting in equal arcs bilaterally. For unilateral disease, this was limited to one 130° arc on the target side. In both cases, each arc would perform a clockwise and counterclockwise rotation.

Patients in the cohort were treated with either 33 or 35 fractions of proton radiation. High-risk targets received 2 Gy/fraction, intermediate-risk targets received 1.8 Gy/fraction, and standard-risk targets received 1.6 Gy/fraction, resulting in a total dose to the targets of 66/70, 59.4/63, and 52.8/56 Gy, respectively. This prescription and fractionation scheme is based on the latest recommendations from the National Comprehensive Cancer Network [37].

For plans with disease extending low into the cervical spine and upper thoracic region, an additional 10° couch kick was used to avoid beams passing through the shoulders.

### 2.3. LET-Based RBE Modeling

Two different models were utilized to calculate the RBE dose, using an in-house script run within the TPS. The first model was the McMahon model [38], defined in Equation (1), which is a simple, linear LET-weighted model:

(1) $${\mathrm{RBE}}_{\mathrm{McMahon}}=1+\kappa \cdot {\mathrm{LET}}_{d}$$

where κ is the empirical fitting parameter with a value of 0.055 µm/keV. The second model, defined in Equation (2), utilized was the McNamara model [30], a more complex model that utilizes tissue-specific α/β ratios:

(2) $${\mathrm{RBE}}_{\mathrm{McNamara}}=\frac{1}{2D_{p}}\left(\sqrt{{\left(\frac{\alpha }{\beta }\right)}_{x}^{2}+4D_{p}{\left(\frac{\alpha }{\beta }\right)}_{x}{\mathrm{RBE}}_{\max}+{4D_{p}^{2}\cdot \mathrm{RBE}}_{\min}^{2}}-{\left(\frac{\alpha }{\beta }\right)}_{x}\right)$$

(3) $${\mathrm{RBE}}_{\max}=0.99064+\frac{0.35605}{{\left(\frac{\alpha }{\beta }\right)}_{x}}{\mathrm{LET}}_{d}$$

(4) $$\mathrm{RB}E_{\min}=1.1012-0.0038703\sqrt{{\left(\frac{\alpha }{\beta }\right)}_{x}}\;\mathrm{LE}T_{d}$$

where D_p_ is the physical proton dose per fraction. RBE_max_ (Equation (3)) and RBE_min_ (Equation (4)) are defined as the asymptotic values of RBE as D_p_ → 0 and D_p_ → ∞, and (α/β)_x_ are the α/β ratios based on x-ray data. In this study, the α/β ratio of nervous tissue was assumed to be 2.5 Gy, reflecting its late-responding, low-proliferation nature [22,39]. In comparison, tumor α/β ratios were assumed to be 10 Gy, consistent with the characteristics of early-responding tissue [40]. This reflects the classical distinction between early- and late-responding tissues: early-responding tissues, such as malignant head and neck tumors, have higher α/β ratios, indicating greater sensitivity to dose per fraction and faster recovery. In contrast, late-responding tissues, such as the spinal cord and brainstem, are more sensitive to fraction size due to their slower cellular turnover.

### 2.4. RBE Enhancement Metric

When analyzing the plan data, we observed that simply adding LET objectives would often modify the physical dose; therefore, merely comparing plan doses within the TPS could be misleading. This led to the establishment of a simple metric to determine the effectiveness of the LET optimization–RBE enhancement–as shown in Equation (5) and defined as the dose-averaged LET (LET_d_)-dependent RBE model dose divided by the RBE 1.1 dose.

(5) $$\mathrm{RBE}\;\mathrm{Enhancement}=\frac{\mathrm{RBE}\;\mathrm{Model}\;\mathrm{Dose}}{\mathrm{RBE}\;1.1\;\mathrm{Dose}}$$

### 2.5. Plan Optimization

All plans were created and optimized in a research version of RayStation (RaySearch Laboratories AB, Stockholm, Sweden). As previously mentioned, for static beam PBS plans, the original plan design remained the same. Still, all patients were reoptimized with an OAR and target objective template to minimize variation in our analysis. This same optimization template was utilized for proton arc plans. Robust optimization of the targets was conducted by incorporating an isotropic shift uncertainty of 3 mm applied individually to each beam. Systematic density uncertainty was set to 3.5% and a maximum allowed statistical uncertainty of 0.5% was utilized. Dose calculations were performed using the Monte Carlo algorithm and a grid resolution of 2 × 2 × 2 mm^3^. After optimization, all plans were compared back to the originally delivered plan to ensure no significant deviations in target coverage. Target coverage was ensured to be equal to or greater than the plans initially delivered to the fifteen patients previously treated.

Though optimization objectives and dose calculation remained the same between the static beam plans and arc plans, adjustments were made to beam computation settings to maximize the effectiveness of the arc plans. It should be noted that dynamic arcs were utilized, rather than discrete. Table 1 outlines the differences in beam computation settings between the static beam PBS and dynamic arc plans. All beam computation settings not listed in Table 1 were kept the same between techniques.

After optimizing each patient’s plan and ensuring plan quality, a copy of the plan was made, and an LET-optimized version was produced for the PBS plan. The original plans, PBS and dynamic arc, as well as the PBS LET optimized plans, were then recalculated and compared with the two RBE models. LET objectives were added to the plans and tested until an optimal, standardized set of objectives was found.

## 3. Results

### 3.1. LET_d_ and Dose Reduction

In many cases, using LET optimization was highly successful, particularly when a critical structure was located near the distal edge of the SOBP.

**Figure 3:**
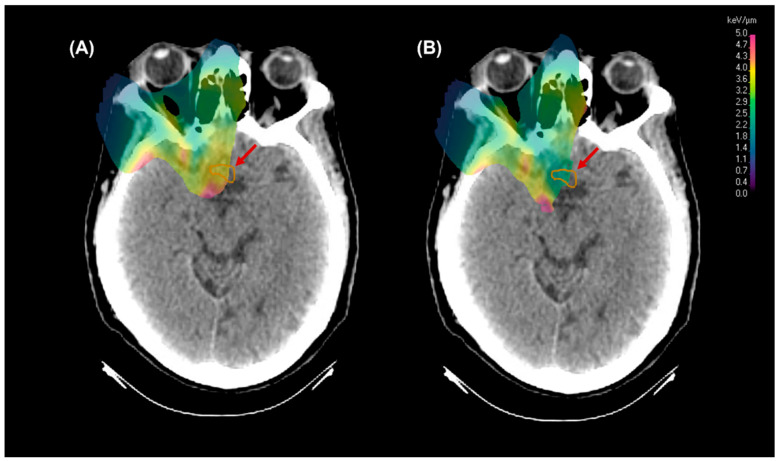
A before (**A**) and after (**B**) visual of the LET distribution demonstrating the shift of a higher LET region in the original plan away from the optic chiasm following LET optimization. The red arrow highlights the particularly critical region of decreased LET distribution in the optic chiasm.

**Figure 4:**
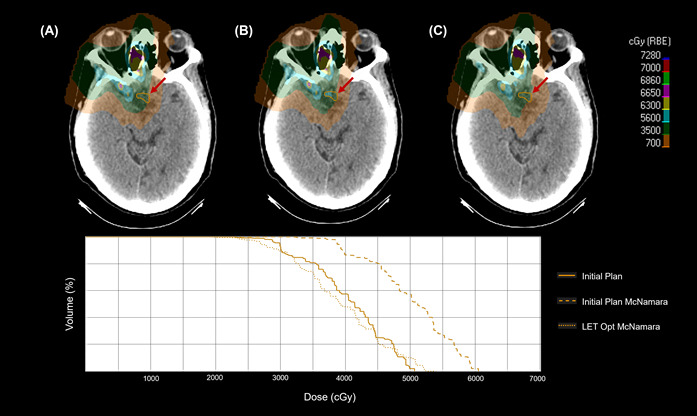
Same patient and axial slice as Figure 3, but demonstrating the change in dose to the optic chiasm for the initial PBS plan (**A**), the initial plan calculated with the McNamara RBE model (**B**), and the LET optimized plan calculated with the McNamara RBE model (**C**). The red arrow highlights the region of concern, and a DVH is below to show the dose differences numerically.

Figure 3 and Figure 4 demonstrate a patient case where LET optimization is critical. Without analyzing the plan dose with a LET-dependent RBE model, one would assume that the maximum dose is well under 60 Gy. After applying the model, the RBE enhancement brings the dose to the optic chiasm above 60 Gy, increasing the risk of toxicity. However, the same model computed on the LET-optimized plan brings the maximum dose back down to near the desired RBE value of 1.1.

### 3.2. RBE Enhancement

**Figure 5:**
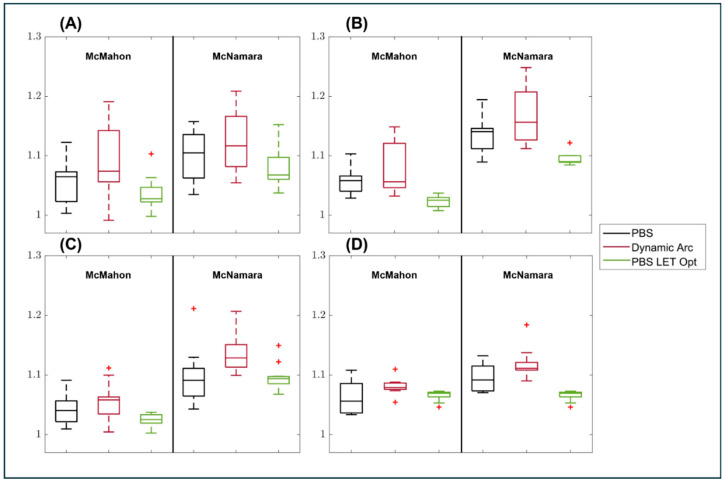
Boxplot display of the RBE enhancement for PBS, dynamic arc, and LET optimized PBS plans (PBS LET Opt). This demonstrates the RBE enhancement for both RBE models for (**A**) brainstem, (**B**) optic chiasm, (**C**) optic nerves, and (**D**) brachial plexus.

RBE Enhancement was previously defined in Equation (5) of Section 2.4. The RBE enhancement was compared for the original PBS plan, the dynamic arc plan, and the LET-optimized PBS plan. The primary concern for nervous tissue toxicity is the maximum dose to the structure; therefore, the maximum dose value was used when calculating the RBE enhancement. The results are presented in Figure 5, and the averages for all structures, as well as both RBE models, are summarized in Table 2.

### 3.3. LET Optimization Variability

While LET optimization has demonstrated benefits and shows an average improvement in RBE enhancement for the structures analyzed, as we can clearly see in Figure 3 and Table 2, there is a very wide range of demonstrated benefits between individual patients. This can be observed by examining the variability in RBE enhancement reduction for one of the structures, the brainstem.

**Figure 6:**
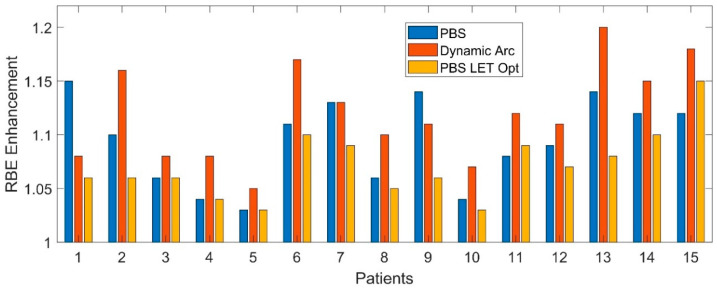
The RBE enhancement from the maximum dose to the brainstem for each patient, as determined by the initial PBS plan, dynamic arc plan, and LET-optimized PBS plan.

Figure 6 illustrates the significant improvements observed among the 15 patients. In multiple patients, there is very little reduction in RBE enhancement. The most significant improvements are observed when there is a high initial RBE enhancement in the PBS plan, whereas patients with lower initial RBE enhancement in the PBS plan do not derive much benefit from LET optimization.

### 3.4. Effective Use of LET_d_ Objectives

One of the primary objectives of this study was to determine an optimal method for utilizing LET optimization in head and neck planning within the TPS. An iterative approach was used to determine the optimal LET_d_ suppression strategy. Initial trials began with maximum LET_d_ caps ranging from 2.0 to 4.0 µm/keV and various dose thresholds for application to the LET objective function. Plans that applied the LET_d_ constraint globally or at dose thresholds below 50% often exhibited degraded target coverage or increased hot spots in surrounding tissue. Conversely, constraints applied only above very high dose thresholds (e.g., 90%) resulted in minimal changes in LET distribution within critical structures.

After systematically testing various combinations across the patient cohort, we found that applying a 2.5 µm/keV LET_d_ constraint above the 80% maximum dose threshold offered the most consistent benefit in reducing RBE enhancement in nervous system structures while preserving overall plan quality. This approach minimized unnecessary penalization of low-dose voxels, which often carry high LET values but are unlikely to contribute meaningfully to biological effect. The selected strategy was applied across the cohort to validate consistency in performance, and it proved robust across varying anatomical geometries and beam arrangements.

**Figure 7:**
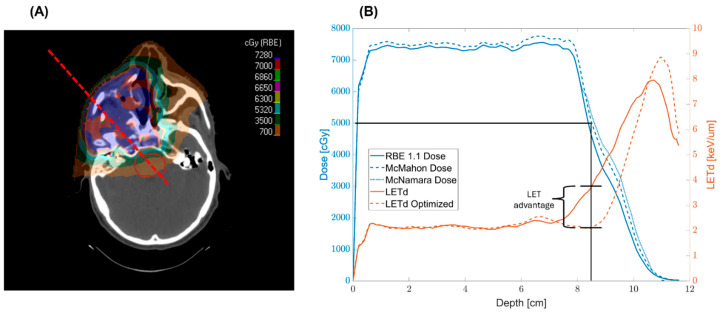
(**A**) shows the trajectory of a line dose profile that travels through the target volume and into the brainstem contour at the distal end of the target. (**B**) displays the line dose and correlated LET_d_ along the same path shown in (**A**). The black line in (**B**) provides a reference point at 50 Gy to assist with visualization, highlighting the LET advantage in the LET optimized plan.

Figure 7 demonstrates the effectiveness of the method previously outlined. The LET optimization did not attempt to modify LET within the target itself, but rather maximized the decrease in LET_d_ in the regions of 40–50 Gy, the level of dose where our concern about high LET is greatest.

## 4. Discussion

### 4.1. LET Distributions & RBE Enhancement

This study evaluated the impact of LET-guided optimization and contributed to the growing body of literature emphasizing the importance of LET-guided planning in proton therapy, particularly in anatomically complex and radiosensitive regions, such as the head and neck. It used two distinct RBE models to assess changes in LET-dependent RBE dose across multiple nervous system structures. While the dosimetric advantages of PBT over photon therapy have been well-established, biological uncertainties associated with spatial variation in LET remain. Numerous studies have consistently demonstrated that ignoring LET heterogeneity may result in localized increases in biological dose that are well above nominal values, potentially leading to unexpected toxicities such as radiation necrosis [41,42]. Wang et al. correlated treatment-induced necrosis with elevated LET regions in pediatric patients undergoing cranial irradiation [43]. Peeler et al. and Handeland et al. confirmed this finding in the context of ependymoma, where LET-dependent biological dose modeling predicted clinical complications, specifically brainstem necrosis, more accurately than constant RBE assumptions [44,45]. These studies underscore the importance of biologically guided plan evaluation, not merely as a research tool, but as a clinical necessity in the delivery of precision treatment.

Our findings support the premise that LET-aware planning can substantially improve the biological safety of treatment plans, especially for healthy tissue and critical structures/OARs located near the distal edge of the SOBP, where LET and, consequently, RBE are at their highest. The RBE enhancement metric we developed—a ratio of LET_d_-weighted RBE model dose to the constant RBE dose of 1.1—proved to be a valuable tool for quantifying the increased risk of toxicity in plans with different optimization strategies.

### 4.2. Dynamic Arc Implications

**Figure 8:**
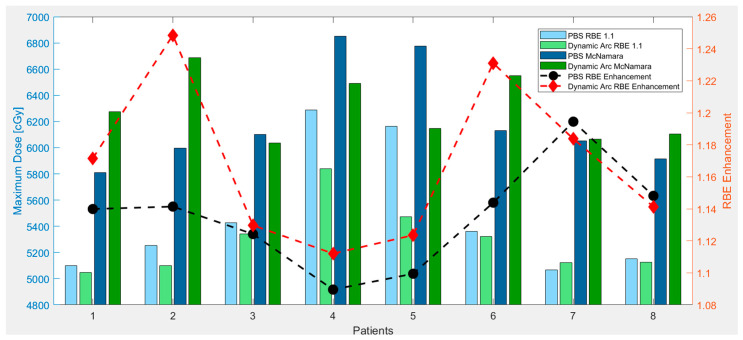
The figure demonstrates the difference between initial plan doses computed with RBE 1.1 and the resulting RBE dose after considering LET-dependent RBE enhancement to the optic chiasm. The RBE 1.1 dose for PBS and dynamic arc is shown next to the resulting McNamara RBE dose for PBS and dynamic arc, along with the RBE enhancement resulting from each delivery method, for each of the eight patients with high doses to the optic chiasm.

The recent emergence of proton arc PAT introduces new degrees of freedom in treatment planning, with studies reporting promising improvements in its conformality and target coverage [33,34,35]. However, the continuous rotation of arc delivery also introduces LET deposition patterns that can differ significantly from static beam approaches. We observed that dynamic arc PBT produced an increase in RBE-weighted dose to critical structures compared to conventional PBS plans. This aligns with emerging concerns in the literature that proton arc techniques may elevate LET at distal edges due to increased path complexity and overlapping beams. Li et al. found that dynamic arc delivery could exacerbate LET heterogeneity in surrounding normal tissue if not adequately accounted for [46]. Our study reinforces these observations, revealing instances where high-LET distributions from the arc beam paths concentrate along shared distal edges of multiple trajectories. For example, under the McMahon model, the RBE enhancement of the optic chiasm rose from 1.05 in PBS to 1.18 in arc plans. Increases were also seen for the brainstem and brachial plexus, with a modest increase for the optic nerves. The McNamara model confirmed this trend with higher average RBE enhancement values for all structures. Figure 8 demonstrates the differences in the RBE 1.1 dose, the McNamara RBE dose, and the levels of RBE enhancement for PBS and dynamic arc delivery.

We can observe the higher RBE enhancement to the optic chiasm for dynamic arc plans, as well as the fact that dynamic arc plans typically have a lower initial RBE maximum dose of 1.1 compared to PBS. However, in multiple cases, the resulting RBE model dose is higher for the dynamic arc than for PBS, despite having a lower RBE dose of 1.1. Although this increase was not drastic in all cases, it highlights the importance of LET considerations in novel delivery techniques such as arc therapy, which are not yet widely studied from a biological dose standpoint.

### 4.3. Impact of LET Optimization

LET optimization successfully mitigated LET-dependent RBE model dose elevations, generally reducing RBE enhancement compared to PBS and dynamic arc plans. In the McMahon model, optic nerves dropped from 1.04 (PBS) and 1.05 (Arc) to 1.02 post-optimization. The brainstem RBE enhancement decreased from 1.05 (PBS) and 1.09 (Arc) to 1.03 post-optimization. The optic nerves also showed improvement, dropping from 1.04 (PBS) and 1.05 (Arc) to 1.02 post-optimization. Using the McNamara model, post-optimization RBE enhancement values were also lower for all four nervous tissue structures, except the optic nerves, which had the same value for PBS and post-optimization PBS plans. However, both were lower than the enhancement in the dynamic arc plans. Overall, the optic nerves showed less RBE enhancement before LET optimization, which is likely due to the optic nerves often being located within the entry dose region, rather than near the distal edge of the beam, unlike the other structures. These reductions in RBE enhancement, although numerically small, are potentially clinically significant, especially when the RBE 1.1 dose is nearing the critical structure tolerances.

### 4.4. Clinical Deployment of LET Optimization

Applying LET optimization objectives provides a means of mitigating elevated RBE doses in critical structures. However, the benefit of LET optimization varied widely across different patients, as indicated in Figure 6 with respect to brainstem RBE enhancement: patients with higher baseline RBE enhancement showed the most significant improvement, while low-risk patients saw limited gains. This inter-patient variability was also noted in earlier studies, highlighting the need for future predictive tools to stratify patients based on their likelihood of LET-related toxicity [9,13,15]. Such variability underscores the importance of pre-optimization risk assessment. A possible direction is to integrate LET-based risk scores or anatomical proximity flags into the treatment planning system to guide when LET optimization is warranted.

LET optimization must be applied judiciously. We found that setting LET_d_ constraints too aggressively or at dose levels too low resulted in a degradation of plan quality, either by underdosing target volumes or compromising nearby critical structures. Lower dose thresholds diverted optimization resources to less relevant regions, while overly strict LET_d_ caps compromised target coverage. Our empirical determination of a 2.5 µm/keV LET_d_ constraint, applied above 80% of the desired maximum dose to the structure, represents a clinically reasonable compromise that maintains target coverage while reducing risk to OARs. This strategy is supported by previous work showing that LET_d_ optimization, when limited to mid-to-high dose regions, avoids unnecessary penalization of low-dose/high-LET voxels that may be biologically irrelevant [9].

Nevertheless, several limitations remain for adopting LET optimization in proton therapy. For example, we had to utilize in-house tools to compute LET-dependent RBE model doses that are not yet available in commercial TPS platforms. Moreover, while two RBE models were employed for comparison, both rely on simplifying assumptions and parameter estimates that may not fully capture the spectrum of in vivo radiobiological responses [47,48]. Additional difficulties, such as the computational burden associated with real-time LET calculations, further complicate clinical use [49]. Yet, significant progress has been made in inverse planning workflows. Utilizing techniques such as nonlinear optimization functions, especially those based on biologically motivated dose surrogates or track-end objectives, can yield plans with improved biological conformity without substantial increases in planning complexity, resulting in superior performance in terms of plan quality and biological relevance, as indicated by multiple studies [28,50]. Further improvements include the selection of beam angles and the use of smaller spot sizes to fine-tune LET distributions [51,52].

Despite these advancements, widely accepted, standardized LET-based constraints are still lacking, and more robust clinical evidence is needed to fully validate these strategies. Prospective validation, with correlational clinical toxicity data, will be essential for transitioning the LET optimization process into routine clinical deployment. Further research should aim to refine LET optimization strategies, investigate their clinical outcomes, and build consensus on standardized implementation. Our findings can contribute to a growing argument for embedding LET objectives directly into inverse planning workflows, showing that LET-based objectives can be standardized and applied efficiently across a cohort of diverse head and neck cases.

## 5. Conclusions

Proton arc therapy, while promising in dose conformality and coverage, may slightly elevate RBE-weighted doses in OARs, emphasizing the need for LET-based assessment and mitigation strategies. LET-guided treatment planning represents a valuable evolution in head and neck proton therapy. Our study demonstrates that integrating a maximum LET_d_ constraint of 2.5 µm/keV, applied above 80% of the desired maximum dose to the structure, offers a practical and effective strategy for reducing RBE-weighted dose to nervous tissue structures, particularly in cases where these structures lie near the distal edge of proton fields. This approach mitigates the potential for radiobiological overdose associated with elevated LET regions, which have been linked to late toxicities such as optic neuropathy, brainstem injury, and brachial plexopathy [17,43,44,45,50]. By implementing LET objectives in plan optimization, we demonstrated a pathway to biologically safer plans without compromising dosimetric quality. Future studies should aim to validate these findings prospectively and explore real-time integration of LET metrics into commercial treatment planning systems. This study demonstrates that with further development and standardization, LET-guided optimization can potentially bridge the gap between the physical precision and the biological uncertainties of tissue response in PBT.

## Figures and Tables

**Table 1:** Differences in beam computation settings for PBS and arc plans.

	Energy Layer Spacing	Spot Spacing	Target Margins (Lateral)
PBS	Automatic with scale = 1	Automatic with scale = 0.7	Automatic with scale = 0.7
Arc	Automatic with scale = 0.25	Constant = 0.6 cm	Constant = 0.5 cm

**Table 2:** Summary of mean RBE enhancement values for the McMahon and McNamara model, for the brainstem, optic chiasm, optic nerves, and brachial plexus.

McMahon:	Brainstem	Optic Chiasm	Optic Nerves	Brachial Plexus
N =	15	8	14	9
**PBS**	1.05	1.05	1.04	1.06
**Dynamic Arc**	1.09	1.18	1.05	1.08
**PBS LET Opt**	1.03	1.02	1.02	1.02
McNamara:	**Brainstem**	**Optic Chiasm**	**Optic Nerves**	**Brachial Plexus**
N =	15	8	14	9
**PBS**	1.09	1.13	1.09	1.09
**Dynamic Arc**	1.12	1.16	1.13	1.11
**PBS LET Opt**	1.07	1.09	1.09	1.06

## Data Availability

Data supporting the findings of this study are available from the corresponding author, Yawei Zhang, upon reasonable request.
